# Supplementary material for: A high-resolution mRNA expression time course of embryonic development in zebrafish
Source: eLife. 2017 Nov 16;6:e30860. doi: 10.7554/eLife.30860 (PMC5690287; doi:10.7554/eLife.30860)
Supplement: Supplementary file 6. [file elife-30860-supp6.zip › biolayout-clusters-files/Cluster125-genes.html]

Cluster125


# Cluster125: Genes

| Ensembl ID | Gene Name | Chr | Start | End | Biotype |
| --- | --- | --- | --- | --- | --- |
| ENSDARG00000002894 | aven | 20 | 29328677 | 29366483 | protein\_coding |
| ENSDARG00000043727 | cwc27 | 10 | 11439873 | 11543186 | protein\_coding |
| ENSDARG00000103127 | gpn2 | 16 | 33984197 | 33989698 | protein\_coding |
| ENSDARG00000053950 | lin37 | 15 | 37688113 | 37698090 | protein\_coding |
| ENSDARG00000015352 | lsg1 | 11 | 33957559 | 33970474 | protein\_coding |
| ENSDARG00000093007 | mphosph6 | 25 | 35447097 | 35451405 | protein\_coding |
| ENSDARG00000068143 | slc39a6 | 2 | 42306868 | 42324566 | protein\_coding |
| ENSDARG00000021404 | zgc:110319 | 13 | 18223678 | 18228604 | protein\_coding |
| ENSDARG00000098992 | zgc:114119 | 19 | 47918634 | 47923366 | protein\_coding |
| ENSDARG00000057100 | zwilch | 18 | 19467582 | 19483580 | protein\_coding |
